# Minimal Hepatic Encephalopathy in Cirrhotic Patients: A New Simple and Fast Digital Screening Method

**DOI:** 10.1002/ueg2.70004

**Published:** 2025-04-15

**Authors:** Henrike Dobbermann, Raffael Schlüter, Yevgeniy Lyubchenko, Johanna Beder, Sven Danneberg, Katharina Mitzlaff, Iris Engelbart, Jakob Jessberger, Felix Braun, Thomas Becker, Christian Labenz, Monique Janneck, Denys Matthies, Friedhelm Sayk, Jens U. Marquardt

**Affiliations:** ^1^ Department of Medicine I University Medical Center Schleswig‐Holstein Lübeck Germany; ^2^ Department of Electrical Engineering and Computer Science Technische Hochschule Lübeck University of Applied Sciences Lübeck Germany; ^3^ Department of General, Visceral‐, Thoracic‐, Transplantation‐ and Pediatric Surgery University Medical Centre Schleswig‐Holstein (UKSH) Kiel Germany; ^4^ Department of Internal Medicine I University Medical Center of the Johannes Gutenberg‐University Mainz Germany; ^5^ Cirrhosis Center Mainz (CCM) University Medical Center of the Johannes Gutenberg‐University Mainz Germany

**Keywords:** app, CHILD, cirrhosis, cognitive deterioration, diagnosis, digital, MELD, psychometric hepatic encephalopathy score, smartphone, Stroop test

## Abstract

**Backround:**

Minimal hepatic encephalopathy is a common and prognostically severe complication of cirrhosis with a significant impact on the quality of life. Detailed diagnostic work‐up of minimal hepatic encephalopathy is time‐consuming and difficult to integrate into daily clinical routine.

**Objective:**

We aimed to develop a new, simple, and easy‐applicable smartphone‐based self‐assessment method for screening of minimal hepatic encephalopathy.

**Methods:**

92 patients with cirrhosis and 20 healthy controls were recruited to perform 3 different short digital tests on smartphones (Tip test (TT), number connection test (dNCT) and modified Stroop test (ST)). Results were correlated with the Psychometric Hepatic Encephalopathy Score (PHES) as the presumed gold standard for minimal hepatic encephalopathy. The impact of age, gender, education, CHILD and MELD scores was further investigated.

**Results:**

All 3 digital tests showed good correlation with PHES (TT *r* = −0.76, dNCT *r* = −0.58, and ST *r* = −0.65; all *p* < 0.001). Digital tests were performed significantly faster (TT median 41s (IQR 36–51s); dNCT median 21s (IQR 8–16s); ST median 76s (IQR 55–99s) than PHES (median 322s; IQR 261–434s); There were significant differences between age groups and different levels of education (*p* < 0.05). AUC for TT was 0.835 (95% confidence interval 0.747–0.922, *p* < 0.001) and highest among all digital tests.

**Conclusion:**

All 3 digital tests proved to be suitable for screening of minimal hepatic encephalopathy. TT showed the highest correlation with reference PHES and was not affected by language skills or color blindness, and, thus, might represent a new and fast method for minimal hepatic encephalopathy detection. Intra‐individually adjusted smartphone‐based thresholds might further eliminate the influence of age, gender, educational level or training, to refine early app‐based alerts in case of cognitive deterioration in cirrhotic patients.

## Graphical abstract

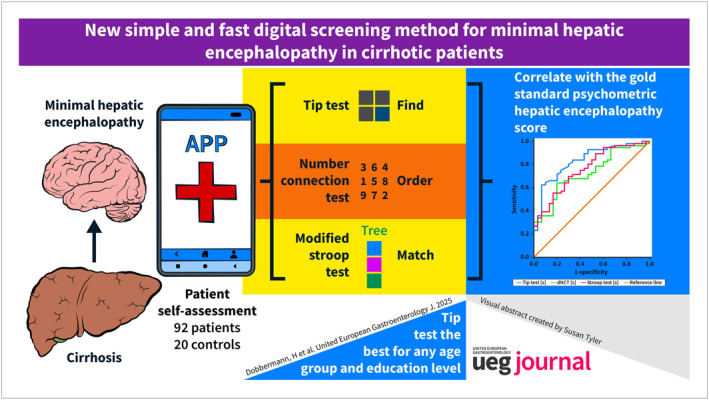

1

**Summary**

- Established knowledge on the subject
  ◦ Minimal hepatic encephalopathy (mHE) is cumbersome to diagnose and often neglected in clinical routine.
  ◦ Established psychometric assessments like Psychometric Hepatic Encephalopathy Score (PHES) paper‐and‐pencil test are time‐consuming, resource‐demanding and not suitable for self‐assessment.
  ◦ Integration of new, smartphone‐based digital methods promises autonomous self‐conducted surveillance with potentially earlier mHE detection.
- Significant and new findings of this study
  ◦ In the present head‐to‐head comparison, three novel smartphone‐based digital tests proved to be rapid and reliable approaches to detect mHE as correlated with concurrent reference PHES.
  ◦ Tip Test showed the best performance. Its pragmatic ease of use, speed, and discriminatory capacity could make it valuable in clinical settings inside and outside the hospital.
  ◦ Potential learning effects at regularly repeated self‐screening and constitutional individual factors warrant adjustment. Predetermined individual baseline and threshold levels based on a smartphone‐app could generate a continuous degradation alert in the event that mHE develops or deteriorates.

## 2 Introduction

Hepatic encephalopathy (HE) is a common and severe complication of cirrhosis, which can significantly affect patients' prognosis [1, 2]. This also applies to minimal hepatic encephalopathy (mHE), the subclinical form of HE [3, 4, 5]. mHE is present in 30%–70% of cirrhosis patients and can significantly impair memory, attention, and reaction time, thereby severely affecting the quality of life [6]. Individuals with mHE typically exhibit prolonged reaction times, which can compromise activities like driving [7, 8]. Furthermore, mHE significantly increases the risk of progression to overt HE (oHE) leading to hospitalizations and higher socioeconomic costs [9, 10, 11]. Diagnosis of mHE presents challenges in clinical practice. The most validated method is the internationally recognized Psychometric Hepatic Encephalopathy Score (PHES), which includes five manual paper‐and‐pencil tests. To perform the test battery usually takes 10–20 min, with an additional 3–10 min for evaluation. Since PHES requires clinical staff and is not suitable for self‐conduct by patients, comprehensive testing of mHE is often neglected in daily clinical routine due to limited resources [12]. This hampers the early detection and treatment of HE [13]. Recently, digital diagnostic tools, such as the critical flicker frequency (CFF) and animal naming test (ANT), have been developed and validated for faster and simpler HE diagnosis [2, 5, 14].

New smartphone‐based methods such as the EncephalApp Stroop Test offer significant advantages compared to conventional methods. This app uses the Stroop effect, a cognitive phenomenon related to inhibitory control [15] where patients have to discriminate between a denominated color and the apparent font color (e.g., the word “blue” displayed in red letters). This multilingual application has been internationally validated as a reliable stand‐alone tool for regular follow‐up testing [16, 17, 18]. Its smartphone compatibility makes it a pragmatic option for detecting mHE in clinical practice, with the potential for wider accessibility in the future. However, the app has some limitations. Results can be severely affected by factors such as age or education level, and it is not suitable for patients who are illiterate or have red‐green color blindness. Additionally, the test can still be time‐consuming and mentally demanding, which might make it frustrating for patients and limit its use as a self‐performed screening tool at home.

Thus, developing a novel, easy‐applicable and fast digital test approach independent of language skills and color vision is highly desirable. Our aim was to develop a practical tool for patient‐based repeated mHE screening at home. As a first step we here report the accuracy of novel digital tests compared to the standard PHES.

## 3 Methods

### 3.1 Study Cohort

Ninety‐two patients with cirrhosis, who were familiar with using a smartphone, were recruited from the hepatology division of our medical department. The diagnosis of liver cirrhosis was established by conventional definition through a combination of imaging techniques (CT, MRI, sonography including transient elastography), laboratory findings (thrombocytopenia and diminished liver synthesis parameters), clinical signs and endoscopic indicators (presence of esophageal varices and signs of portal hypertensive gastropathy) or liver biopsy. To stage the severity of liver cirrhosis,‐Pugh score and Model of end‐stage liver disease (MELD) were calculated. We excluded patients who either did not provide consent, showed cognitive impairments indicative of a manifest neurological disorder or overt HE (≥ 2, West Haven classification), or had taken sedative medications (e.g. benzodiazepines or narcotics) or alcohol on the day of testing. Recruitment was performed during regular outpatient visits. A subset of the study group received inpatient care at our university hospital due to cirrhosis‐related complications other than overt HE. 20 healthy control subjects without any history of liver disease were recruited from the municipal normal population.

### 3.2 Testing for Hepatic Encephalopathy

All patients underwent a clinical examination by a hepatologist to rule out overt hepatic encephalopathy, acute intoxication, or acute withdrawal symptoms prior to inclusion. This was complemented by laboratory tests, to control for confounding metabolic disturbances. Subsequently, patients and controls performed the PSE test battery, developed by Weissenborn et al. (version 2019) to obtain the individual PHES [12, 19]. Test results were compared with PSE‐inherent reference values tailored to age, gender, and years of school education. A score lower than −4 is considered pathological, indicating mHE [12].

Following PHES assessment, participants underwent three smartphone‐based tests: the Tip Test (TT), digital Number Connection Test (dNCT), and the modified Stroop test (Stroop test).

1. *Tip Test:* On the smartphone display, a grid pattern of 70 tiles was presented. Next to 69 gray tiles, one tile was distinctively colored in blue. Once the participant tapped this blue tile, its position in the grid changed. Participants were requested to tap this alternating blue tile as fast as possible, the whole task consisted of 40 taps, and the time needed for the whole run was recorded.
2. *dNCT:* Inspired by the Number Connection Test A from the PHES, the dNCT was adapted for the smartphone display. Due to the smaller screen size, it showed only 9 numbers scattered randomly across the screen. Participants had to connect these numbers in ascending order by “swiping” their finger, thus also including a motoric component akin to the line drawing test of PHES. The time needed to complete the task and finger movements were recorded.
3. *Modified Stroop test:* The test described hereafter examines inhibitory control similar to the EncephalApp by Bajaj et al. In an attempt to create a faster, but still cognitively challenging test, we chose to limit the total number of iterations (30 items compared to at least 140 items for “On” and “Off” state of EncephalApp) and, to retain complexity, included five different colors instead of the original three. Patients had to identify the font color of a word and select the corresponding color out of one of five buttons displayed at the screen's bottom (white font on a blue background) (See Figure S1). However, the word itself denominated a different color (e.g. the word “green” displayed in blue font, thus, the “blue” button should be selected). Both the time required and the number of incorrect selections was recorded.

Screenshots of the three digital tests described above are depicted in Figure S1.

### 3.3 Statistical Data Analysis

The results from the smartphone‐based tests for cirrhosis patients and controls were correlated with the corresponding individual total PHES results. Additionally, the results were correlated with the subject's age, educational level, as well as MELD and Child Score. Moreover, the results of digital tests were sub‐analyzed for effects of gender, age, and educational level.

To determine the normal distribution of metric data from the smartphone‐based tests, the Shapiro‐Wilk test was performed. In cases where data were not normally distributed, Spearman's rank correlation method was applied for correlation analyses and data were displayed as the median and inter‐quartile range (IQR). Statistical significance of results across various groups was analyzed using the‐U test. All tests were two‐tailed, and a *p*‐value ≤ 0.05 was considered to be statistically significant.

To examine the diagnostic accuracy of the digital TT, dNCT and Stroop test, a ROC (Receiver Operating Characteristic) curve analysis was conducted. Cut‐off values were determined by calculating Youden's Index (J) as well as the point closest top left (C) by using the formulas listed below and choosing the highest and lowest values as a suitable cut‐off value.

J=sensitivity+specifity−1

$$C=\sqrt{{\left(1-\text{sensitivity}\right)}^{2}+{\left(1-\text{specifity}\right)}^{2}}$$

All statistical evaluations were performed using SPSS software, Version 28.0 (SPSS IBM Corp., New York, USA).

### 3.4 Ethics

This study was approved by the ethics committee of the University of Lübeck (file number 2022–556). Written informed consent was obtained from all participants prior to testing.

## 4 Results

### 4.1 Baseline Characteristics

**FIGURE 1:**
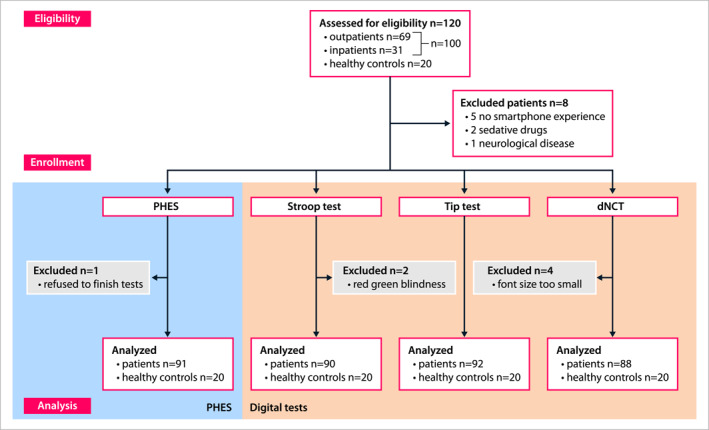
Flow diagram of eligible and enrolled or excluded participants (cirrhotic patients and healthy controls) for PHES (left side, blue) and digital mHE‐tests (right side, orange). dNCT: digital number connecting test; PHES: psychometric hepatic encephalopathy score.

A total of 100 patients with cirrhosis were screened for eligibility. Eight patients had to be excluded due to missing smartphone experience (*n* = 5), intake of sedative medication (*n* = 2) or underlying neurological conditions (*n* = 1) as predefined. Finally, 92 patients with cirrhosis were enrolled comprising 35 females (38%) and 57 males (62%) with an average age of 60 ± 9 years (range: 38–87 years). 67 patients were recruited from the outpatient department and 25 were inpatients. The majority of the patients were classified as compensated cirrhosis Child stage A (*n* = 55; 59.8%). The flow chart of eligibility and enrollment for mHE testing is displayed in Figure 1. As a control cohort, 20 healthy individuals (9 males) with a mean age 56 ± 16 years (range: 26–76) were additionally recruited. 20 patients (21.7%) consumed alcohol on a regular basis, 9 (9.8) within the last week, but not within 24 h prior to mHE testing, and two patients used other drugs (cannabis *n* = 1, benzodiazepine *n* = 1), occasionally, but not within the last 48 h. Baseline characteristics of patients and controls are detailed in Table 1.

**TABLE 1:** Baseline characteristics of the total cohort of patients with cirrhosis and subgroups with and without minimal hepatic encephalopathy (mHE) according to PHES or of healthy controls.

Variable	Total number of patients *n* = 92	Without mHE *n* = 31 (34%)	mHE *n* = 60 (66%)	Healthy controls
Male *n* (%)	57 (62)	17 (55)	40 (67)	9 (45)
Female *n* (%)	35 (38)	14 (45)	20 (33)	11 (55)
Age in years, mean ± SD	60 ± 9	57 ± 9	62 ± 10	56 ± 16
Education in years, mean ± SD	9.8 ± 1.4	10.2 ± 1.6	9.8 ± 1.3	12.2 ± 1.4
Outpatient number *n* (%)	67 (73)	27 (87)	39 (65)	
Inpatient number *n* (%)	25 (27)	4 (13)	21 (35)	
Etiology of cirrhosis *n* (%)				
Alcohol	57 (62)	22 (71.0)	35 (58.3)	
Alcohol + hepatitis B/C	6 (6.6)	1 (3.2)	5 (8.3)	
Metabolic	13 (14.1)	2 (6.5)	11 (18.4)	
Viral hepatitis (B + C)	3 (3.3)	1 (3.2)	1 (1.7)	
Autoimmune (PBC/AIH)	6 (6.5)	2 (6.5)	4 (6.7)	
Other	7 (7.6)	3 (9.7)	4 (6.7)	
Child pugh score, mean ± SD	6 ± 1.6	6 ± 1	7 ± 2	a
A *n* (%)	55 (59.8)	27 (87)	27 (45)	
B *n* (%)	34 (37)	4 (13)	30 (50)	
C *n* (%)	3 (3.3)	0 (0)	3 (5.1)	
MELD score, mean ± SD	11 ± 5	9 ± 4.6	12 ± 5.1	a
MELD Na score, mean ± SD	13 ± 6	10 ± 4.8	14 ± 1.7	a
Laboratory values mean ± SD				
Bilirubin μmol/L	23.3 ± 31.7	23.5 ± 46.4	23.4 ± 21.2	
Creatinine μmol/L	103.7 ± 71.3	81.7 ± 25.7	116 ± 84	
Albumin mg/L	36.5 ± 7.3	41.1 ± 6.2	34 ± 6.5	
INR	1.2 ± 0.3	1.2 ± 0.4	1.3 ± 0.3	
Thrombocytes/nL	159.9 ± 84.8	160.4 ± 78	157.4 ± 87.7	
Na mmol/L	137.2 ± 4.8	138.9 ± 2.9	137 ± 5.3	
Extent of ascites *n* (%)				
None	63 (68.5)	27 (87)	35 (58.3)	
Mild	13 (14.1)	3 (9.7)	10 (16.7)	
Moderate to severe	16 (17.4)	1 (3.2)	15 (25)	
Prior decompensations *n* (%)				
Ascites	59 (64.1)	19 (61.3)	40 (66.7)	
SBP	14 (15.2)	1 (3.2)	13 (21.7)	
Eos. varices	55 (59.8)	20 (64.5)	35 (58.3)	
Variceal bleeding	14 (15.2)	3 (9.7)	11 (18.3)	
HRS	12 (13)	3 (9.7)	9 (15)	
oHE	9 (9.8)	1 (3.2)	8 (13.3)	
cHE	11 (12)	3 (9.7)	8 (13.3)	
Alcohol consumption in the last 3 months *n* (%)	20 (21.7)	6 (19.4)	13 (21.7)	
Alcohol consumption in the last 7 days *n* (%)	9 (9.8)	4 (12.9)	5 (8.3)	
Consumption of > 24 g (men) or 12 g (women) alcohol per day *n* (%)	4 (4.5)	2 (6.5)	2 (3.3)	
Reason for hospitalization *n* (% of inpatients)				
Hydrope decomp.	11 (44)	1 (25)	10 (47.6)	
Icteric decomp.	1 (4)	1 (25)	0 (0)	
SBP	1 (4)	0 (0)	1 (4.8)	
Varic. ligation	2 (8)	1 (25)	1 (4.8)	
Other infections	4(16)	0 (0)	4 (19.1)	
Anemia	2 (8)	1 (25)	1 (4.8)	
Pulmonary embolism	1 (4)	0 (0)	1 (4.8)	
Unspecific	3 (12)	0 (0)	3 (14.3)	

*Note:* Unspecific: contains stomach pain, exsiccosis.

Abbreviations: decomp., decompensation; Eos., esophageal; SBP, spontaneous bacterial peritonitis; varic., variceal.

^a^ *p* < 0.001 for comparison of patient subgroups with versus without mHE.

### 4.2 Test Results of PHES and Smartphone‐Based Tests

In order to document present individual cerebral performance, all participants underwent the standardized and validated PSE‐test to obtain the PHES, which comprises five subtests and is the conventionally accepted gold standard for mHE detection [19]. One patient declined to perform the “line tracing” subtest and was excluded from further analysis.

According to the PHES, mHE was confirmed in 60 of the 92 patients (65%) and was ruled‐out in all healthy controls as expected. To complete the PHES assessment (combined durations of the five subtests, excluding the time taken for explanations and evaluation of test results), patients required a median time of 322 s (IQR 261–434 s) and healthy individuals about 314 s (median, IQR 302–361 s).

Subsequently, participants performed the three digital tests. All 92 participants of the patient cohort completed the TT. Two of the 92 subjects were excluded from the Stroop test due to red‐green color blindness. Moreover, the results of the first four participants for the dNCT had to be excluded, because the initially chosen font size of 30 pt proved too small for appropriate smartphone screen visibility. After adjusting the font size to 50 pt, the test was well applicable to the remaining 88 participants. Missing cases were excluded from the respective correlation analyses (Figure 1). All healthy control subjects completed the digital tests.

**FIGURE 2:**
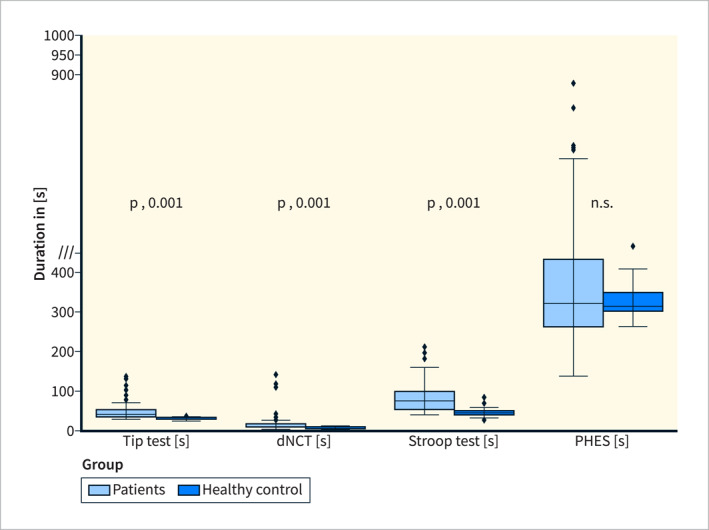
Box‐plots of time (seconds) required to perform each of the three smart‐phone based mHE tests in comparison to the time needed for PHES testing (time required for explanation and evaluation of PHES not included) in cirrhotic patients versus healthy controls.

To perform the digital TT, cirrhotic patients required a median time of 41 s (IQR 36–51 s), while healthy controls needed about 31 s (median, IQR 28–31 s). The dNCT patients required a median of 21 s (IQR 8–16 s) and healthy individuals 7 s (median, IQR 5–9 s). In the patient cohort, the median of time required to complete the modified Stroop test was 76 s (IQR 55–99 s) and for control subjects 46 s (IQR 40–49 s). The respective boxplots of results are shown in Figure 2.

### 4.3 Differences Between Patients With and Without mHE

**FIGURE 3:**
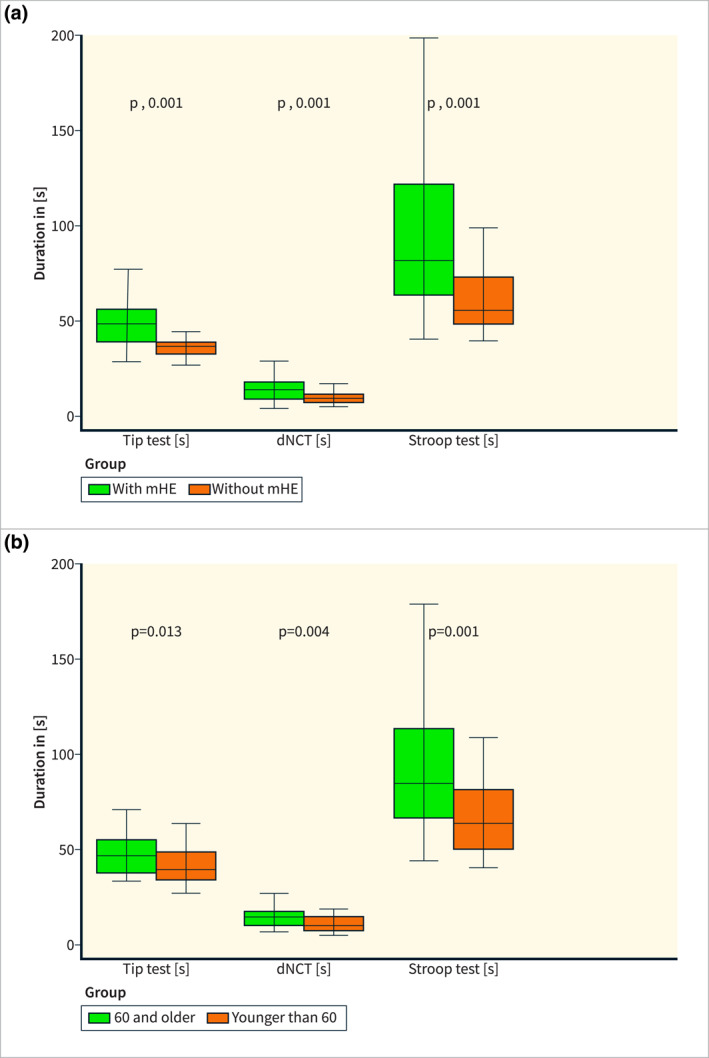
Box‐plots comparing time required to perform digital Tip test, Stroop test, and dNCT (a) in cirrhotic patients with versus without mHE and (b) for the patient cohort dichotomized at the 50th percentile into age group < 60 years versus ≥ 60 years.

As expected, there were marked differences in digital test results between the patient subgroup diagnosed with mHE by PHES and those without PHES (all *p* < 0.001); results are detailed in Table 2 *and* depicted in Figure 3a. Patients with mHE had a rate of about three errors (median, IQR 1–8). In contrast, patients without mHE only made about one mistake (median, IQR 0–2.5). Notably, since each error has to be corrected instantaneously by the subject, higher error rate results in a longer duration to complete the test. Therefore, test duration is the primary read‐out combining the speed of performance and error rate.

**TABLE 2:** Test duration in seconds (median; IQR) of the three digital tests in subgroups of cirrhotic patients according to presence of mHE and dependence on age or level of education.

Cirrhotic patients	Without mHE	With mHE	*p*	≤ 60 years	> 60 years	*p*	Education < 10 years	Education ≥ 10 years	*p*
	(*n* = 60)	(*n* = 32)		(*n* = 46)	(*n* = 46)		(*n* = 42)	(*n* = 48)	
Tip Test	37 (33–39)	48 (39–57)	< 0.001	39 (33–48)	47 (37–55)	0.005	45 (37–52)	39 (33–49)	0.033
dNCT	10 (7–12)	14 (9–18)	< 0.001	9 (7–15)	14 (10–17)	< 0.001	15 (11–18)	10 (7–14)	< 0.001
Modified stroop	56 (48–76)	82 (62–124)	< 0.001	64 (50–81)	85 (66–119)	0.002	95 (63–126)	66 (50–81)	0.002

### 4.4 Correlation Between PHES and Digital Test Results

**FIGURE 4:**
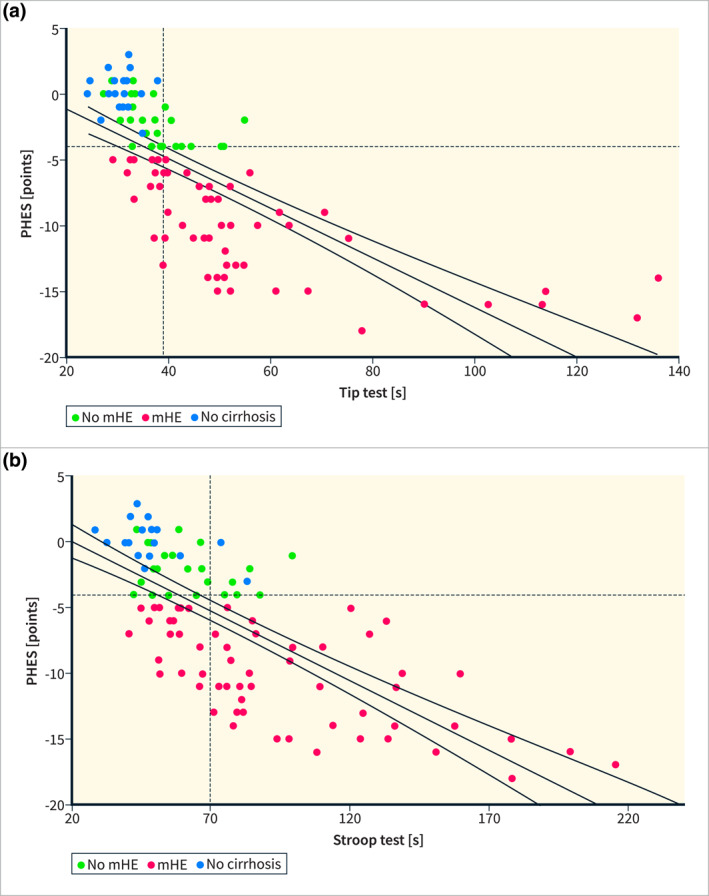
Scatter plot and linear regression (CI 95%) of the (a) digital Tip Test (TT) and (b) Stroop Test with reference to PHES with a PHES of < −4 defining mHE (depicted by the horizontal dotted line) Green dots depict cirrhotic patients without mHE, red dots cirrhotic patients with mHE and blue dots healthy controls without mHE. In accordance with the point closest to the top left at ROC analysis (Figure 6), a cut‐off duration of about 39 s for the TT (depicted by the vertical dotted line) yielded a sensitivity of 75% with specificity of about 76%, and of about 70 s for the modified Stroop Test (sensitivity 70%, specificity 72%).

The digital test results were thoroughly evaluated by within‐subject comparison with the composite total PHES. In cirrhotic patients, all digital tests demonstrated statistically significant (*p* < 0.001) correlations with the PHES. Notably, in this cohort, the TT showed the strongest correlation (*r* = −0.76), followed by the Stroop test (*r* = −0.67) and the dNCT (*r* = −0.59). In healthy controls, in contrast, no significant correlations were observed between the cumulative PHES and the digital tests (Figure 4 a and b).

### 4.5 Correlation Between Digital Test Results and Clinical Characteristics

**FIGURE 5:**
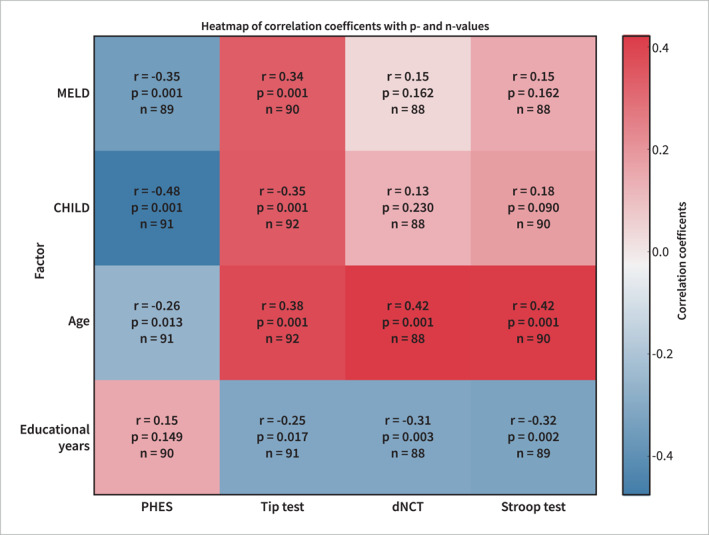
Correlation coefficients (r) for the PHES and the 3 digital tests in cirrhotic patients with regard to scores of disease severity (MELD, Child Pugh), age and years of school education. Blue: negatively correlated, red: positively correlated, intensity of color: strength of correlation.

When correlating MELD and Child Score with the PHES and digital test results, positive coefficients with high statistical significance were found for the PHES (*p* < 0.001) and for the TT (*p* < 0.001), but not for the dNCT or Stroop Test. Age (all *p* < 0.001) and educational level (TT *p* = 0.017, dNCT p0.003, Stroop test *p* = 0.002), in contrast, showed a significant correlation with the results of all digital tests (*p* < 0.05). Both are well‐acknowledged influencing factors for the PHES, and, therefore, standard evaluation of the PHES is already adjusted to age and educational level. Consistently, the correlation of the PHES with age was only weak in our patient cohort (*r* = −0.259; *p* = 0.013) and there was no correlation with years of school education (*r* = 0.153; *p* = 0.149) (Figure 5).

In our cohort of healthy control subjects, there was a positive correlation between age and the results of each digital test (TT *p* = 0.004, dNCT *p* < 0.001, Stroop test *p* = 0.002). However, educational level showed no significant correlation since there was only one individual with less than 10 years of schooling. As expected, results of the age‐ and education‐adjusted PHES did not correlate with these parameters.

### 4.6 Detailed Analysis of the Impact of Age and Educational Level on Digital Test Results

In order to further characterize the potential impact of age as an influential factor on the results of the smartphone‐based tests, the patient cohort was divided into two subgroups based on the 50^th^ percentile, that is individuals of ≤ 60 years and > 60 years (each group *n* = 46). For all three tests, the median time to perform the digital tests was significantly shorter in the younger compared to the older subgroup (TT *p* = 0.005; dNCT *p* < 0.001; Stroop test *p* = 0.002). Results are reported in detail in Table 2 and depicted in Figure 3b.

Overall, healthy controls performed the smartphone‐based tests faster than our patient cohort (all *p* < 0.001) (Figure 2). Likewise, to the cirrhotic patient cohort, those healthy individuals aged less than 60 years (*n* = 10) were significantly faster than healthy individuals aged 60 years or older (*n* = 10) (TT *p* = 0.005; dNCT *p* < 0.001; Stroop test *p* = 0.002).

To explore the potential influence of educational level on the outcomes of the smartphone‐based tests, participants were subgrouped based on the duration of their school education: individuals with 6–9 years were compared to those with 10 or more years of formal education. We chose this categorization in analogy to the subgroups of the PHES. Subjects with longer education completed all three digital tests significantly faster compared with those with less education (TT *p* = 0.033; dNCT *p* < 0.001; Stroop test *p* = 0.002) (Table 2). As expected, there was no statistically significant difference between the two patient subgroups based on their educational levels in PHES (−7.8 vs. −6.7 points; *p* = 0.328). For the cohort of healthy controls, we could not well divide into two subgroups because there was only one individual with an educational level of < 10 years.

Furthermore, we found no significant gender‐related differences in the digital test results between males and females.

### 4.7 Sensitivity and Specificity

**FIGURE 6:**
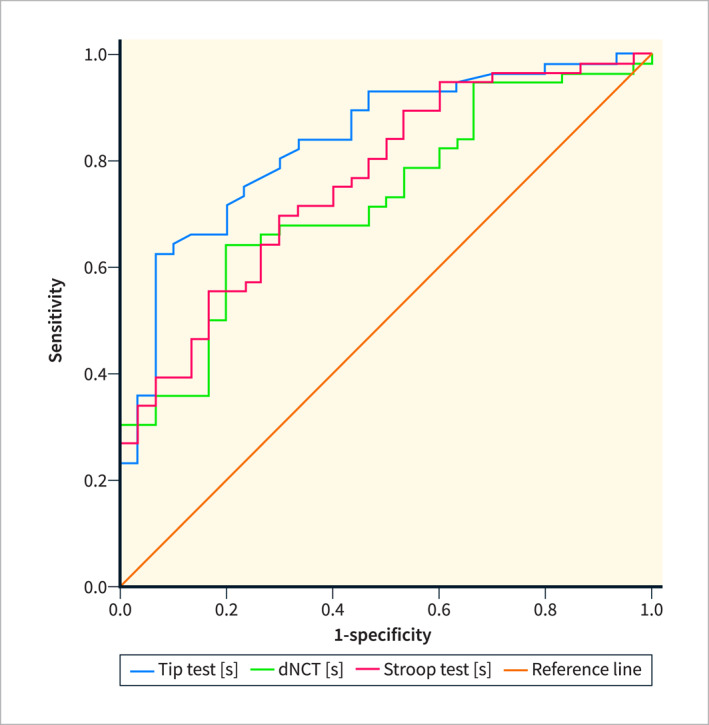
Receiver operating characteristic (ROC) curves for the digital mHE tests (Tip test: AUC 0.832. dNCT: 0.738 and Stroop test: AUC 0.779) with reference to the PHES as diagnostic standard.

Additionally, the diagnostic accuracy of the smartphone‐based digital tests was examined in relation to the PHES using Receiver Operating Characteristic (ROC) curve analysis. There was an AUC of 0.832 (95% confidence interval (CI) 0.742–0.921, *p* < 0.001) for the TT, an AUC of 0.738 (95% CI 0.632–0.845, *p* = 0.001) for the dNCT, and an AUC of 0.762 (95% CI 0.680–0.878, *p* < 0.001) for the Stroop test (Figure 6). For the TT, using Youden's Index, we calculated an optimal cut‐off value of 44,5s resulting in a sensitivity of 63% and a specificity of 93%. In our clinical setting, we felt that a higher sensitivity at the cost of specificity was warranted and thus calculated the point closest to the top left, resulting in a cut‐off value of 39 s with a corresponding sensitivity of 75% and specificity of 76%.

## 5 Discussion

Diagnostic work‐up of subclinical mHE in cirrhotic patients is highly relevant but time consuming and staff intensive, and thus difficult to integrate into clinical routine when assessed by standardized psychometric paper‐and‐pencil tests. Digital methods promise autarkical self‐assessment with potentially earlier mHE detection. Hence, smartphone‐based mHE surveillance might save time and staff resources. In this study, we present three simple smartphone‐based digital mHE tests all showing good correlations with the PHES, which is the internationally accepted diagnostic standard for mHE detection.

On a closer view, the Tip test (TT) demonstrated the highest correlation coefficients with the PHES. In contrast to the PHES and Stroop test, it is independent of native language, literacy level and color vision, allowing widespread applicability. Furthermore, it is independent of red‐green color‐blindness, which further enhances its universal use. The TT in particular received favorable feedback from patients (data not shown). Our modified Stroop test demonstrated short testing times with still acceptable correlations to PHES. Recently, Bajaj et al. have also addressed the issues of test duration and ease of use in their improved “Quick Stroop” version, featuring only the easier “Off” State of their test [20]. Our third test, the dNCT, showed the weakest correlations and AUC. This mirrors the low sensitivity of NCT A and B as stand‐alone tests as described by Weissenborn et al. in their original characterization of PHES [12]. Therefore, the dNCT does not appear promising as a future stand‐alone testing method.

In our healthy control subjects, an excellent performance at digital tests was in concordance with normal PHES‐results. Since these controls showed no signs of cognitive impairment, there was little variation in test results in both PHES and digital testing. Due to this rather uniform performance without substantial scattering, we found no correlation between digital tests and PHES, as expected.

In patients with cirrhosis, digital tests discriminated well between patients with and without mHE and correlated significantly with MELD and Child–Pugh scores. These findings are plausible as severe cognitive impairment is well‐described in patients with advanced liver disease [2, 21]. In cirrhotic patients, multiple comorbid factors might additionally influence the development of mHE. In our cohort, for example, chronic alcohol consumption was found to be the leading etiology of cirrhosis. Underlying neurotoxic damage might interfere with smartphone‐based mHE self‐assessment, but should likewise confound the PHES and therefore not affect our correlation analysis [21].

Apart from clinical confounders and medication, constitutional factors such as ethnicity, age, gender, or education might influence mHE assessment. The PHES already acknowledges this by adjusted reference values [19]. In digital testing, age emerged as an important determinant in both patients and controls. This was most evident in all digital tests (*p* < 0.05) when comparing patients aged ≤ 60 with those > 60 years, representing the 50^th^ percentile of our study population, emphasizing age as a substantial determinant. It's worth noting that in addition to a confounding effect, advanced age can elevate the risk of mHE, leading to a higher prevalence in older patients [6]. To further explore age‐related effects, future studies should aim at deriving age‐adjusted thresholds for better digital mHE discrimination. Still, the fact that the present non‐age‐adjusted version of our digital tests did correlate well with the age‐corrected PHES obviously argues in favor of its strong value independent of age.

Educational level exhibited a significant correlation with the digital test results in our patient cohort. Notably, for the TT, this aspect had only weak statistical significance. In our healthy controls, we did not observe any significant correlation, which might be attributed to the homogeneity of educational levels in this cohort. The PHES, already adjusted for education, showed no such disparities [19]. In addition, evaluation of the PHES is gender‐specific. For the digital tests, our patient cohort did not show any gender‐related differences. However, the gender imbalance in our cohort of patients with mHE, with male participants outnumbering females (*n* = 40 vs. 20), might limit statistical accuracy and warrant approval in larger cohorts.

### 5.1 Perspective

The present work reports the head‐to‐head comparison of smartphone‐based digital tests with concurrent mHE assessment by PHES as the well‐acknowledged gold standard. At this level, the digital tests proved to be a rapid and reliable patient‐based approach to detect mHE. Their ease of use, speed, and discriminatory capacity could make them particularly valuable in clinical settings inside and outside the hospital. In particular, the TT emerges as an attractive and pragmatic, language and color‐neutral screening tool. Potential learning effects of regularly repeated self‐screening as well as constitutional individual factors warrant consideration. To address these caveats, it would be conceivable to determine individual baseline and threshold levels based on a smartphone‐app. Subsequently, this app could generate a continuous degradation alert in case mHE develops or deteriorates. This should prompt consultation at the hepatology outpatient clinic at an early stage, to check for the need of interventions and avoid hospitalization. While the present pilot‐trial aimed at characterizing the novel digital test strategies for mHE detection as compared to the PHES, our findings warrant confirmation and further adjustment in larger longitudinal trials. As next steps these should aim (1) at defining age‐adjusted reference margins for the digital tests, (2) at investigating their suitability for regular self‐surveillance in everyday life and (3) at validating their diagnostic efficacy and clinical prognostic value. Further studies should implement individualized adaptations, to improve independent and regular self‐surveillance, thereby facilitating early and patient‐refined mHE management with improved prognostic outcome.

## Conflicts of Interest

J.U.M. and F.S. received honoraria from Merz and abbvie, which were not related to the current study. No conflicts of interest, financial or otherwise, are declared by the other authors.

## Supporting information

Figure S1

## Data Availability

The data that support the findings of this study are available from the corresponding author upon reasonable request.
