# Supplementary material for: Minimal Hepatic Encephalopathy in Cirrhotic Patients: A New Simple and Fast Digital Screening Method
Source: United European Gastroenterol J. 2025 Apr 15;13(7):1194–204. doi: 10.1002/ueg2.70004 (PMC12463687; doi:10.1002/ueg2.70004)
Supplement: Supplementary file 1 — Figure S1 [file UEG2-13-1194-s001.pptx]

## Slide 1
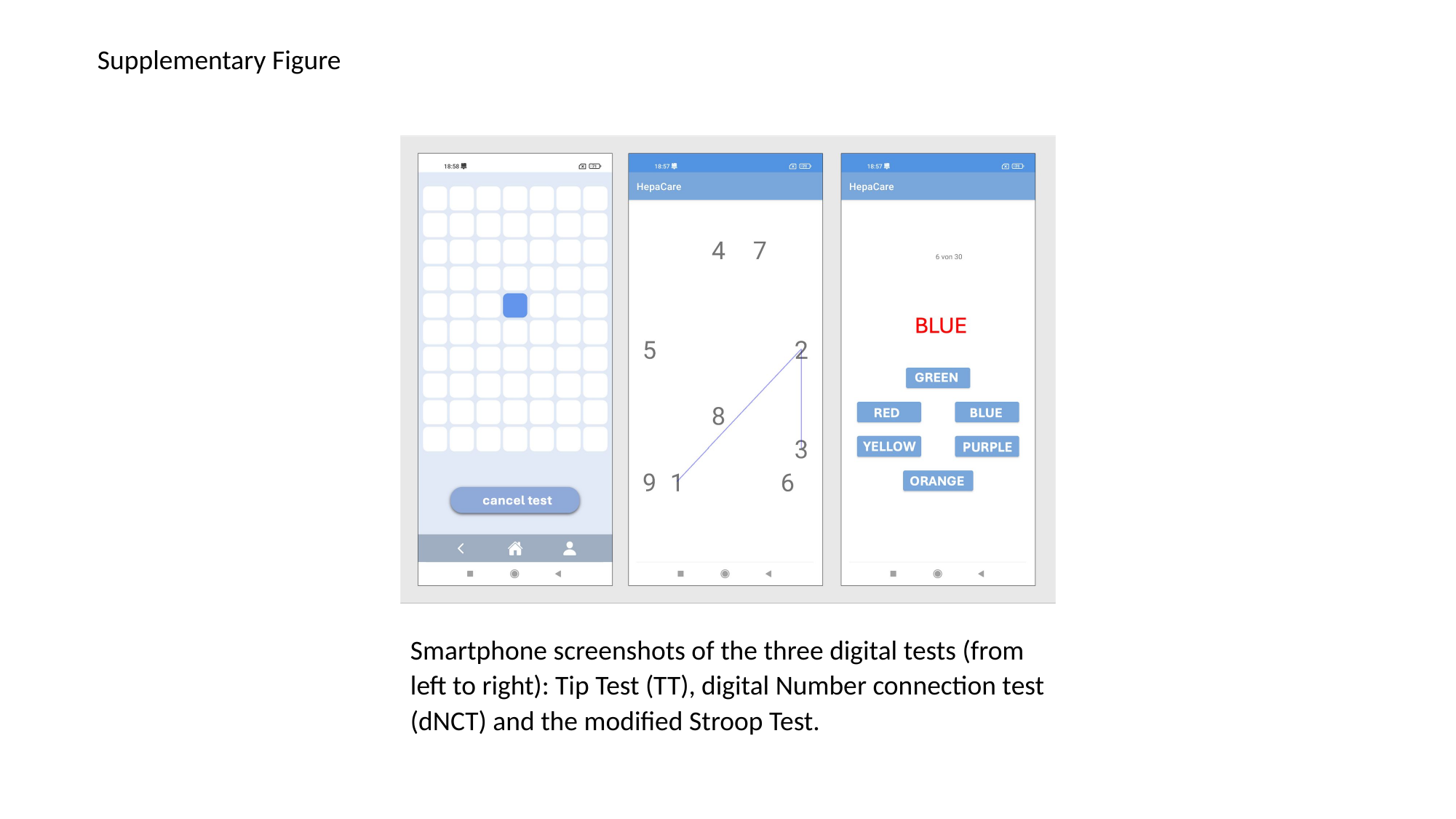

Supplementary Figure
Smartphone screenshots of the three digital tests (from left to right): Tip Test (TT), digital Number connection test (dNCT) and the modified Stroop Test.
